# Optimizing the synthesis and purification of MS2 virus like particles

**DOI:** 10.1038/s41598-021-98706-1

**Published:** 2021-10-06

**Authors:** Khadijeh Hashemi, Mohammad Mahdi Ghahramani Seno, Mohammad Reza Ahmadian, Bizhan Malaekeh-Nikouei, Mohammad Reza Bassami, Hesam Dehghani, Amir Afkhami-Goli

**Affiliations:** 1 Division of Biotechnology, Faculty of Veterinary Medicine, Ferdowsi University of Mashhad, Mashhad, Iran; 2 Department of Basic Sciences, Faculty of Veterinary Medicine, Ferdowsi University of Mashhad, Mashhad, Iran; 3 Institute of Biochemistry and Molecular Biology II, Medical Faculty of the Heinrich-Heine University, Düsseldorf, Germany; 4 Program in Genetics and Genome Biology, Hospital for Sick Children, Toronto, ON Canada; 5 Nanotechnology Research Center, Institute of Pharmaceutical Technology, Mashhad University of Medical Sciences, Mashhad, Iran; 6 Stem Cell Biology and Regenerative Medicine Research Group, Research Institute of Biotechnology, Ferdowsi University of Mashhad, Mashhad, Iran

**Keywords:** Biological techniques, Biotechnology, Chemical biology, Drug discovery

## Abstract

Introducing bacteriophage MS2 virus-like particles (VLPs) as gene and drug delivery tools increases the demand for optimizing their production and purification procedure. PEG precipitation method is used efficiently to purify VLPs, while the effects of pH and different electrolytes on the stability, size, and homogeneity of purified MS2 VLPs, and the encapsulated RNA sequences remained to be elucidated. In this regard, a vector, capable of producing VLP with an shRNA packed inside was prepared. The resulting VLPs in different buffers/solutions were assessed for their size, polydispersity index, and ability to protect the enclosed shRNA. We report that among Tris, HEPES, and PBS, with or without NaNO3, and also NaNO3 alone in different pH and ionic concentrations, the 100 mM NaNO3-Tris buffer with pH:8 can be used as a new and optimal MS2 VLP production buffer, capable of inhibiting the VLPs aggregation. These VLPs show a size range of 27-30 nm and suitable homogeneity with minimum 12-month stability at 4 °C. Moreover, the resulting MS2 VLPs were highly efficient and stable for at least 48 h in conditions similar to in vivo. These features of MS2 VLPs produced in the newly introduced buffer make them an appropriate candidate for therapeutic agents’ delivery.

## Introduction

Delivering small organic or inorganic molecules into the biological systems for therapeutic and research purposes has always been a challenge for biologists. In addition to natural barriers challenging the reach of the target organ and further, inside the cells, the stability of the material of interest to be delivered and that of the delivering agent, plus their safety and toxicity levels introduce additional limitations and challenges. In this line, various approaches such as those involving nanoscale platforms have been tested to find optimal delivery agents^1,2^. Viruses have naturally evolved optimal and efficient targeted cargo delivery and, hence, have successfully been used by biologists for the same purposes. However, nearly all of the naturally occurring viruses that may be most suitable for a specific cargo delivery purpose are also pathogenic and/or posing other threats. Therefore, various modifications are applied to a virus before it could safely be used as a vector. Virus-like particles (VLPs), which are frequently used by biologists for targeted and efficient cargo delivery, are viral particles normally devoid of considerable pathogenicity^3,4^. The MS2 VLP is a 27 nm spherical RNA bacteriophage virus-like particle, consisting of 180 identical 129-amino acid coat protein subunits of ~ 14 kDa that form the icosahedral face for a T = 3 surface lattice^5–7^. MS2 VLP due to its small size and special shape, as well as its ability to encapsulate different nucleic acids, proteins, and other small molecules, and its endocytic route of entry has been considered a suitable vector for delivering into and tracking cargoes in the targeted cells^8^. The coat proteins of MS2 VLP can be recombinantly expressed and assembled in bacteria and yeasts^9–12^. It is conveniently prepared, packaged, delivered, and has high safety margins. Thus, the MS2 VLP has been used as a potential medicinal carrier^13^ for the treatment of various diseases such as prostate cancer^14^, hepatocellular carcinoma^15,16^, and other diseases^8,17–20^. It has also been used for various research purposes including the investigation of RNA trafficking in live cells^21^, in platforms of vaccines and peptide epitope identification^22–27^, and quality control of DNA and RNA virus detection^28–30^.

It is well characterized that nucleic acids and other small molecules can be packaged in the MS2 VLP via their interaction with a short RNA stem-loop structure (19 nucleotides: *pac* site), which in turn binds to coat protein dimers^5,31–33^. Thus, the interaction with the 5’-terminus of the so-called *pac* site has been used as a preferred strategy to encapsulate different cargoes^2,8^. In this respect, it is very important to understand the behavior and assembly of virus-like particles and their aggregation and stability in different conditions. To make efficient VLPs, various physicochemical factors such as pH, temperature, and ionic strength of the solution, the ideal size, homogeneity, and stability of produced VLPs, and their capability to protect cargoes from degradation need to be taken into account. Likewise, in the recombinant production of MS2 VLPs, upon the transformation of plasmids expressing MS2 coat protein and a part of maturase into the bacteria, the most appropriate pH and buffers to support the stability and homogeneity of VLPs are sought after^12,34–36^.

Different studies have shown that the amounts of electrolytes are important factors for the assembly of MS2 bacteriophage and related VLPs^8,9,35^. On the other side, if we consider the pH_c_ as a critical pH value of the buffer used for the production of particles, the values below and above pH_c_ may lead to repulsive electrostatic interactions and stable suspension of VLPs^9,37^. It has been shown that MS2 VLP particles remain in the detached form at 100 mM NaNO3 concentration of buffer solution, and the isolated forms of VLPs in low ionic strength (1 mM and 10 mM) of NaNO3 buffer were merely seen at a pH value near the isoelectric pH (between 3 and 4)^9^. In this study, we introduce a new and optimized buffer to prepare the most stable condition for VLPs. To simulate the in vivo conditions, we assess the stability of VLPs produced in this buffer after incubation in 50% FBS and at 37 °C temperature. We report improved buffer conditions that are used for the preparation of VLPs with appropriate size, shape, and stability.

## Results

### NaNO3-Tris (100 mM, pH:8) buffer provides the best condition for shape, particle size consistency, and dispersity of VLPs

To confirm the expression of the coat protein of VLPs, ~ 14 kDa disassembled protein of purified MS2 VLPs was separated by electrophoresis on 12.5% acrylamide gel and detected using specific antibody and Western blot method. BL21 (DE3) bacterial lysate showed both monomers and dimers of coat protein (Supplementary Fig. S1).

**Figure 1:**
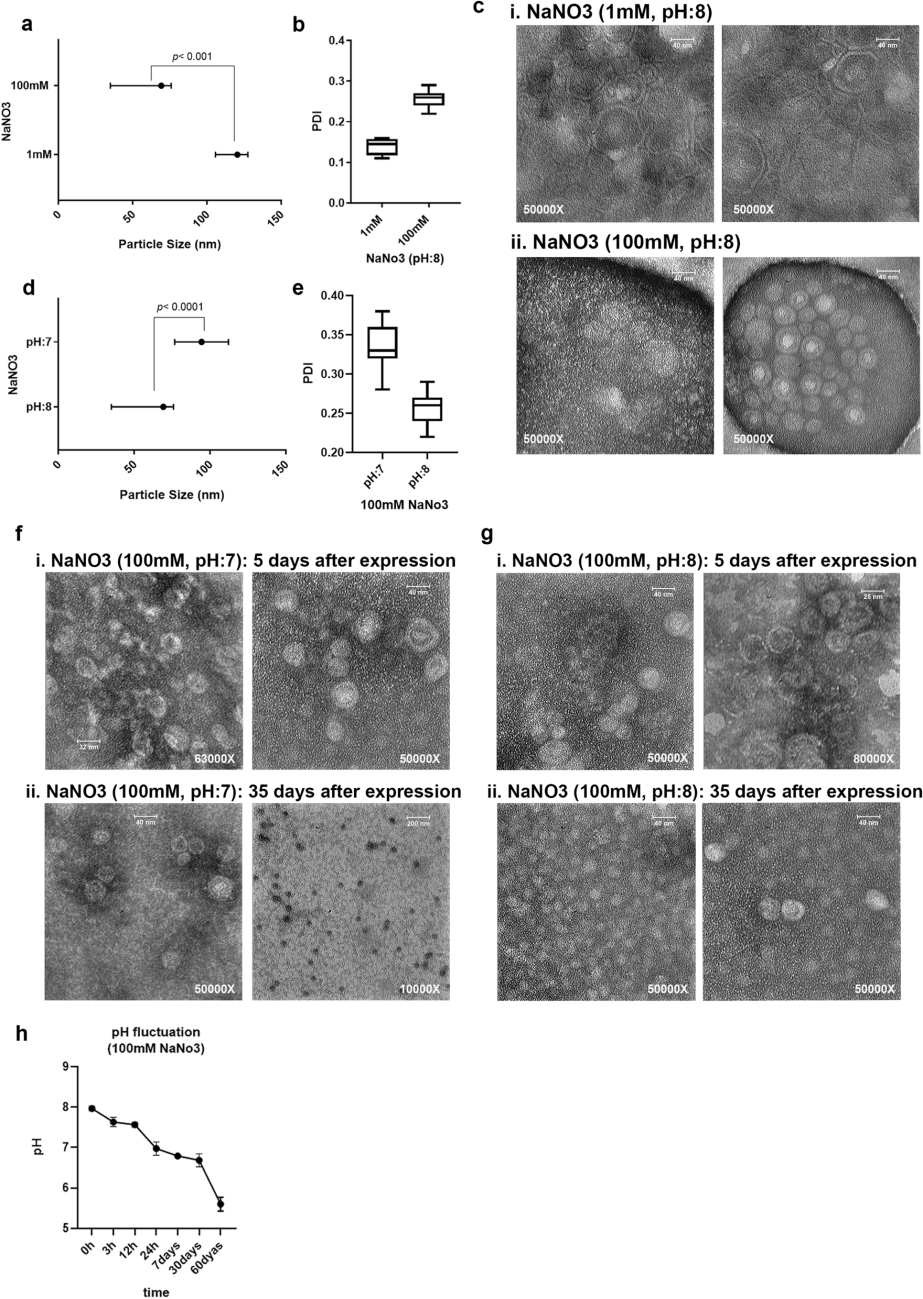
NaNO3 solution is the optimized NaNO3 solution for VLP purification during the PEG precipitation method. (**a**) The particle size of MS2 VLPs produced in 1 mM and 100 mM NaNO3 solution (pH:8). Results are shown as median with range, p < 0.001. (**b**) Polydispersity Index (PDI) shows the homogeneity of VLPs in different NaNO3 concentrations. (**c**) Verification of size and shape of VLPs by TEM. Images were taken from different parts of slides, confirming the differences in shape and size. i) VLPs in 1 mM NaNO3 solution ii) VLPs in 100 mM NaNO3 solution (pH:8). (**d**) The particle size of MS2 VLPs in 100 mM NaNO3 solution (pH:7 and 8). Results are expressed as median with range, p < 0.0001. (**e**) Polydispersity Index (PDI) of VLPs in different pH: 7 and 8. (**f**,**g**) Verification of size and shape of VLPs prepared in 100 mM NaNO3 solution (pH:7) and 100 mM NaNO3 solution (pH:8) by TEM, respectively. (**i**) 5 days after VLP expression. ii) 35 days after VLP expression. (**h**) pH variation of 100 mM NaN03 during 60 days. pH:8 at the first time point was considered.

The influence of different NaNO3 concentrations (1 mM and 100 mM, pH:8) and pH values (7 and 8) were assessed in MS2 VLP expression, production, and purification by the PEG precipitation method. The reduction of NaNO3 concentration could increase the particle size to larger than 100 nm (*p* < 0.001, Fig. 1a), probably because of PEG aggregation around the MS2 VLPs or agglomeration of the VLPs. PDI values in the 100 mM NaNO3 solution were lower than the values in the 1 mM NaNO3 solution (Fig. 1b). While TEM results indicated the size and shape variation in the 1 mM NaNO3 solution, the MS2 VLPs in the 100 mM NaNO3 solution disclosed much more shape uniformity. The 1 mM concentration of NaNO3 induced MS2 VLPs deformation (Fig. 1c).

Decreasing the pH of 100 mM NaNO3 solution from 8 to 7 drastically increased the average size of particles (Fig. 1d, p < 0.0001). Also, the PDI values were increased in lower pH (Fig. 1e). TEM analysis of VLPs at different time points (during 35 days after VLP preparation) indicated a reduction in the size and an increase in the uniformity of VLPs when the 100 mM NaNO3 solution (with pH 7 or 8) was used (Fig. 1f, g). Additionally, more negative zeta potential values were registered in 100 mM NaNO3 (pH:7) compared to 100 mM NaNO3 (pH:8) (Supplementary Fig. S2). To investigate the cause of these changes, the pH of NaNO3 solution was evaluated through different time points during a 60 days period which showed the reduction of pH value from 8 to 5.5 (Fig. 1h). pH values below 6, resulted in instability and dissociation of MS2 particles.

**Figure 2:**
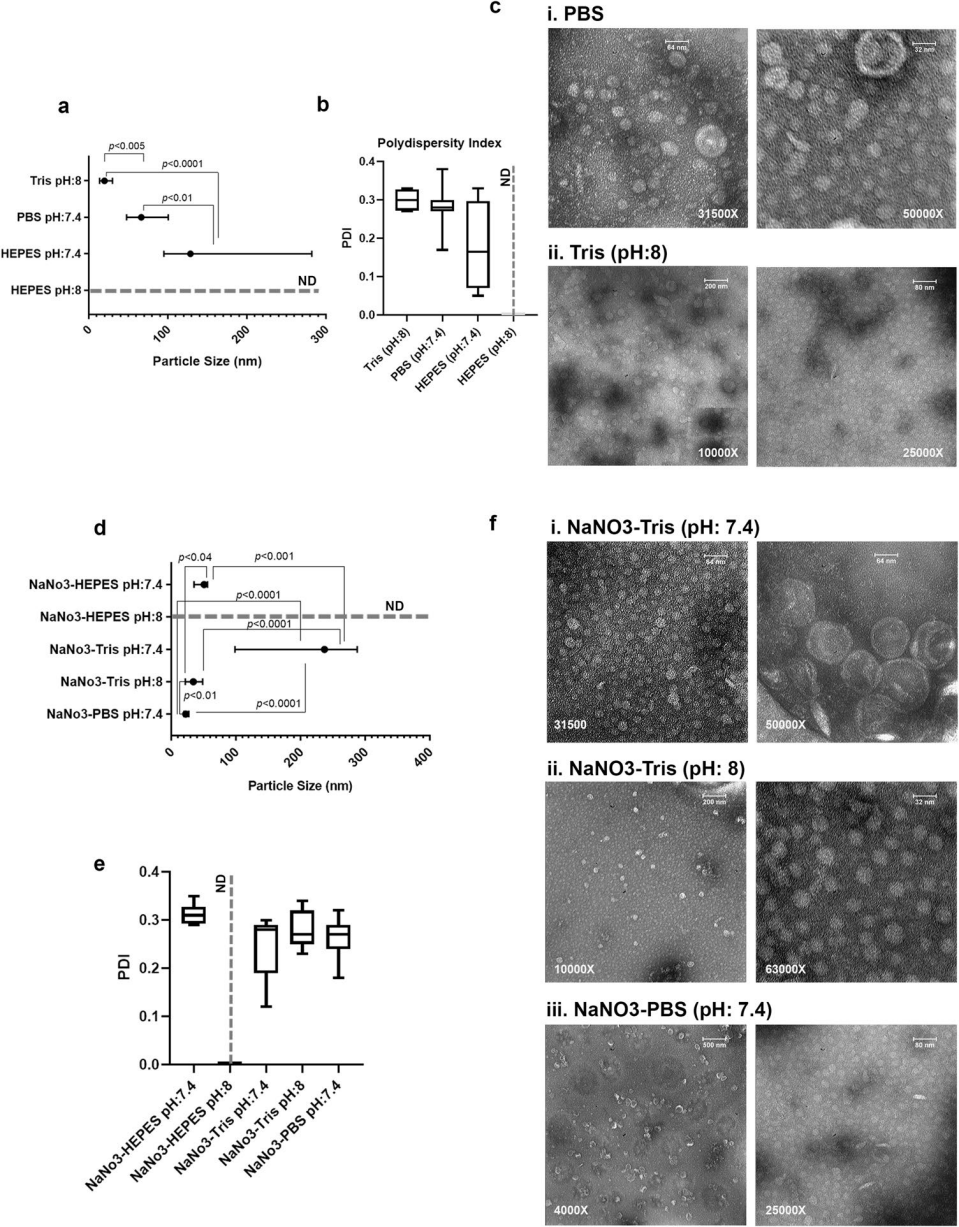
VLPs produced in NaNO3-Tris buffer showed the best shape, particle size consistency, and dispersity. (**a**) The size was detected in Tris (pH:8), PBS (pH:7.4), HEPES (pH:7.4), and HEPES (pH:8). The size of VLPs in HEPES (pH:8) was out of range (> 1000 nm), and not detectable (ND). Results are shown as median with range, p < 0.01, p < 0.005, and p < 0.0001. (**b**) Polydispersity Index (PDI) of VLPs in different Buffers. (**c**) VLP suspension in i) PBS (pH:7.4) and ii) Tris (pH:8). Particles below 100 nm were observed by TEM. (**d**) The particle size of VLPs prepared in 100 mM NaNO3 mixed with buffers. The size of particles in NaNO3- HEPES (pH:8) was extra-large, out of range (> 1000 nm), and not detectable (ND). Results are shown as median with range, p < 0.04, p < 0.01, p < 0.001, p < 0.0001. (**e**) The PDI value of various VLP suspensions was measured. ND: not detectable. (**f**) VLP suspensions with a size below 100 nm were investigated by TEM. i) VLPs in NaNO3-Tris (pH:7.4), ii) VLPs in NaNO3-Tris (pH:8), iii) VLPs in NaNO3-PBS (pH:7.4).

Considering the severe pH fluctuation of NaNO3 solutions, during MS2 VLP production and purification, different buffers were used to stabilize the pH-dependent changes in particle size and PDI. As shown in Fig. 2a and b, the type of buffer had a significant effect on the particle size and PDI. While particle size and PDI of VLPs produced in HEPES (pH: 8) were higher than 1 µm (out of range of detection), lowering the pH to 7.4 could reduce the size and expand its PDI variation between 0.05 to 0.33. TEM analysis was performed on samples with particles smaller than 100 nm in size. The results showed more homogeneous particles in Tris buffer (pH:8) compared to particles in PBS buffer (pH:7.4) (Fig. 2c, and Supplementary Fig. S3b).

To find a more effective and stable production buffer for PEG precipitation of VLPs, solutions of NaNO3 (100 mM) in Tris (1 M, pH: 7.4 and 8), HEPES (pH: 7.4 and 8), and PBS (pH:7.4) were examined. In general, NaNO3 addition to various buffers boosted the VLP preparation efficiency. Although the particles in NaNO3-HEPES (pH:7.4) were smaller than particles in NaNO3-HEPES (pH:8) (extremely large, out of range of detection), yet the size of particles in NaNO3-HEPES (pH:7.4) was bigger than the size of the native MS2 bacteriophage (27–30 nm) (Fig. 2d). The average PDI value was about 0.31 for particles in NaNO3-HEPES (pH:7.4). In this case, suspensions of VLPs with particle sizes below 100 nm were observed by TEM microscopy. Additionally, VLPs produced in NaNO3-Tris (pH:7.4) were much bigger than particles in NaNO3-Tris (pH: 8) (*p* < 0.0001). VLPs in NaNO3-PBS (pH:7.4) displayed a much smaller size than the native MS2 bacteriophage (27-30 nm). PDI measurement of VLPs produced in NaNO3-Tris (pH:7.4) showed a wide size distribution in comparison to VLPs in NaNO3-Tris (pH: 8), which was also confirmed by TEM analysis (Fig. 2e and f). This discrepancy might be explained by the VLP instability in a narrow pH range, so that only small differences in pH may lead to MS2 VLP aggregation. Zeta potential could not be evaluated because of the buffering nature of HEPES, PBS, and Tris (Supplementary Fig. S2).

**Figure 3:**
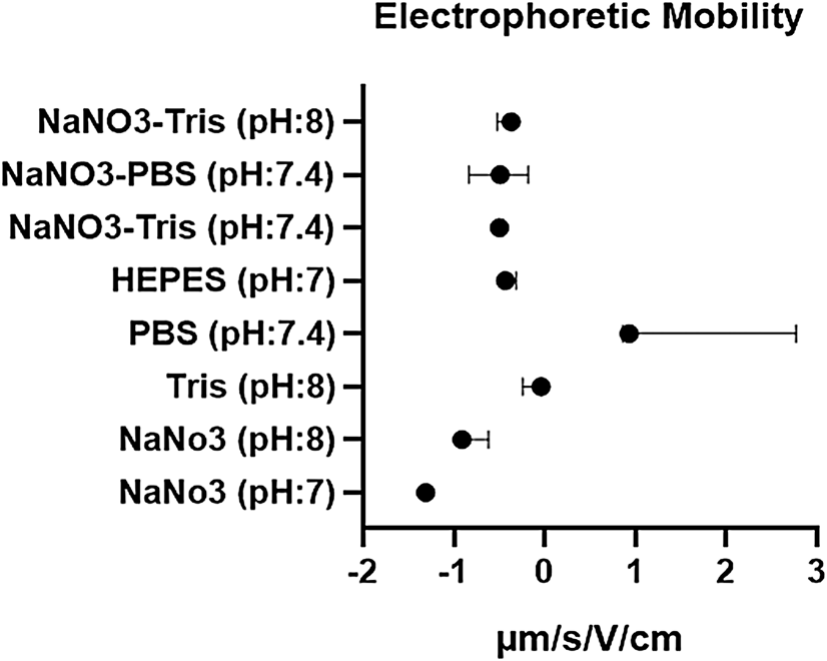
Electrophoretic mobility (µ) of MS2 VLPs in different solutions and buffers. *µ* value of MS2 VLPs was negative in all solutions and buffers except the VLPs in PBS (pH:7.4). Samples were diluted at 1:10 in selected buffers and solutions. Data were shown as median with range.

Electrophoretic mobilities measured for VLPs purified in different solutions and buffers showed the function of the components and also the effect of the pH of these solutions and buffers. As displayed in Fig. 3, the electrophoretic mobilities (*µ*) of MS2 VLPs were negative in all solutions and buffers except for the VLPs in PBS (pH:7.4). Results were shown the variable electrophoretic mobility for VLPs in PBS alone or with 100 mM NaNO3 (Fig. 3). Quantitatively, the average of this mobility for VLPs in NaNO3 (pH:7)(− 1.3 µm/s/V/cm) is more negative than VLPs in NaNO3 (pH:8) (− 0.81 µm/s/V/cm). Although this assessment is about − 0.1 µm/s/V/cm for VIPs in Tris (pH:8) buffer, this amount is more negative for 100 mM NaNO3-Tris (pH:7.4) (− 0.5 µm/s/V/cm) and 100 mM NaNO3-Tris (pH:8) (− 0.43 µm/s/V/cm).

### MS2 Virus-like particles produced in the NaNO3-Tris (pH:8) were more stable than VLPs produced in the NaNO3 solution (pH:8)

**Figure 4:**
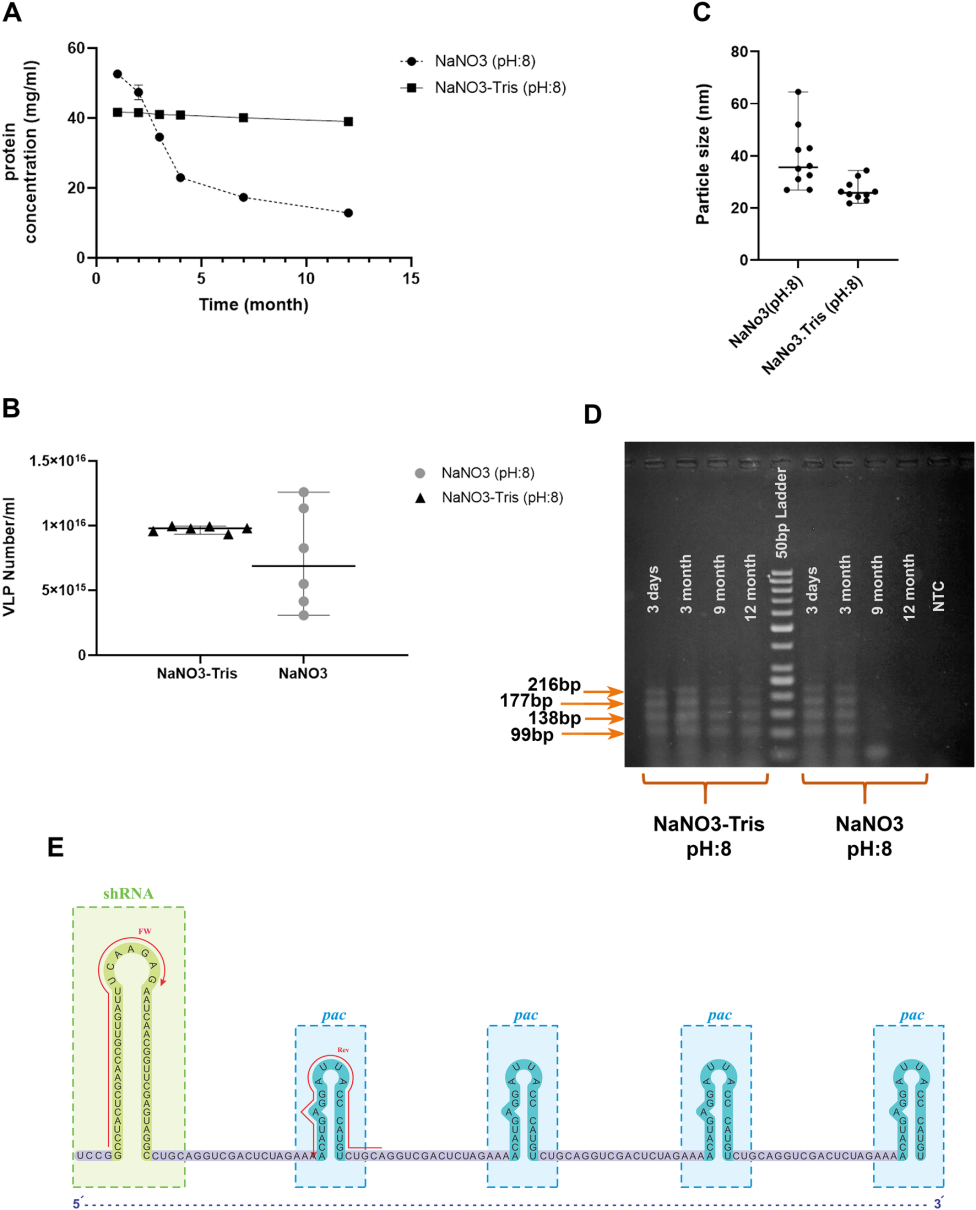
VLPs produced in the NaNO3-Tris were more stable than VLPs produced in the NaNO3 solution. (**a**) Protein concentration evaluation of VLP suspension in two buffers. The amount of protein was reported as mg ml-1. (**b**) Number of VLPs in 1 ml VLP suspension according to the protein concentration. The values are from repeated measurements of samples through 12 months. (**c**) The scattered plot of VLP size in NaNO3 solution and NaNO3-Tris buffer (100 mM NaNO3 and pH: 8, for both of them). The size was analyzed 10 times in each sample and was shown as median with range. (**d**) Gel electrophoresis for RT-PCR fragments (99, 138, 177, and 216 bp); RT-PCR fragments of RNA extracted from VLPs in NaNO3-Tris (pH: 8)- left, and VLPs in NaNO3 (pH: 8)- right, after 3dyas, 3, 9, and 12 months. (**e**) The sequence of shRNA and four hairpins (*pac* sites) was packed in MS2 VLPs. Primer locations were shown as Fw and Rev on the sequence. The stability of VLPs and their ability to protect the inner shRNA was checked by RNA extraction and RT-PCR.

To assess the stability of VLP coat proteins in NaNO3 (100 mM, pH:8) solution and NaNO3-Tris (100 mM, pH:8) buffer, protein concentration was measured by spectrophotometry. Protein concentration and the number of VLPs in NaNO3 (pH:8) solution decreased over time (1, 2, 3, 4, 7, and 12 months), while it was stable for VLPs in NaNO3-Tris (pH:8) (≈40 mg ml^−1^, ≈9e + 15) (Fig. 4a and b). Moreover, particle size assessment of VLPs in two different solutions noticeably indicated the narrow and remarkably homogeneous distribution of VLPs produced in NaNO3-Tris (pH:8) buffer (Fig. 4c, Supplementary Fig. S3a).

Considering the RNA sequence packed in MS2 VLPs (Fig. 4e), we performed RT-PCR to evaluate the stability of these RNAs in NaNO3 (pH:8) and NaNO3-Tris (pH:8) solutions. Four amplicons (99, 138, 177, and 216 bp) were detected on 2% agarose gel. These amplicons for VLPs generated in NaNO3-Tris (pH:8) buffer were detectable for 12 months, while for VLPs produced in NaNO3 solution they were detected for 3 months (Fig. 4d).

### VLPs prepared in NaNO3-Tris buffer remained stable in the presence of serum

**Figure 5:**
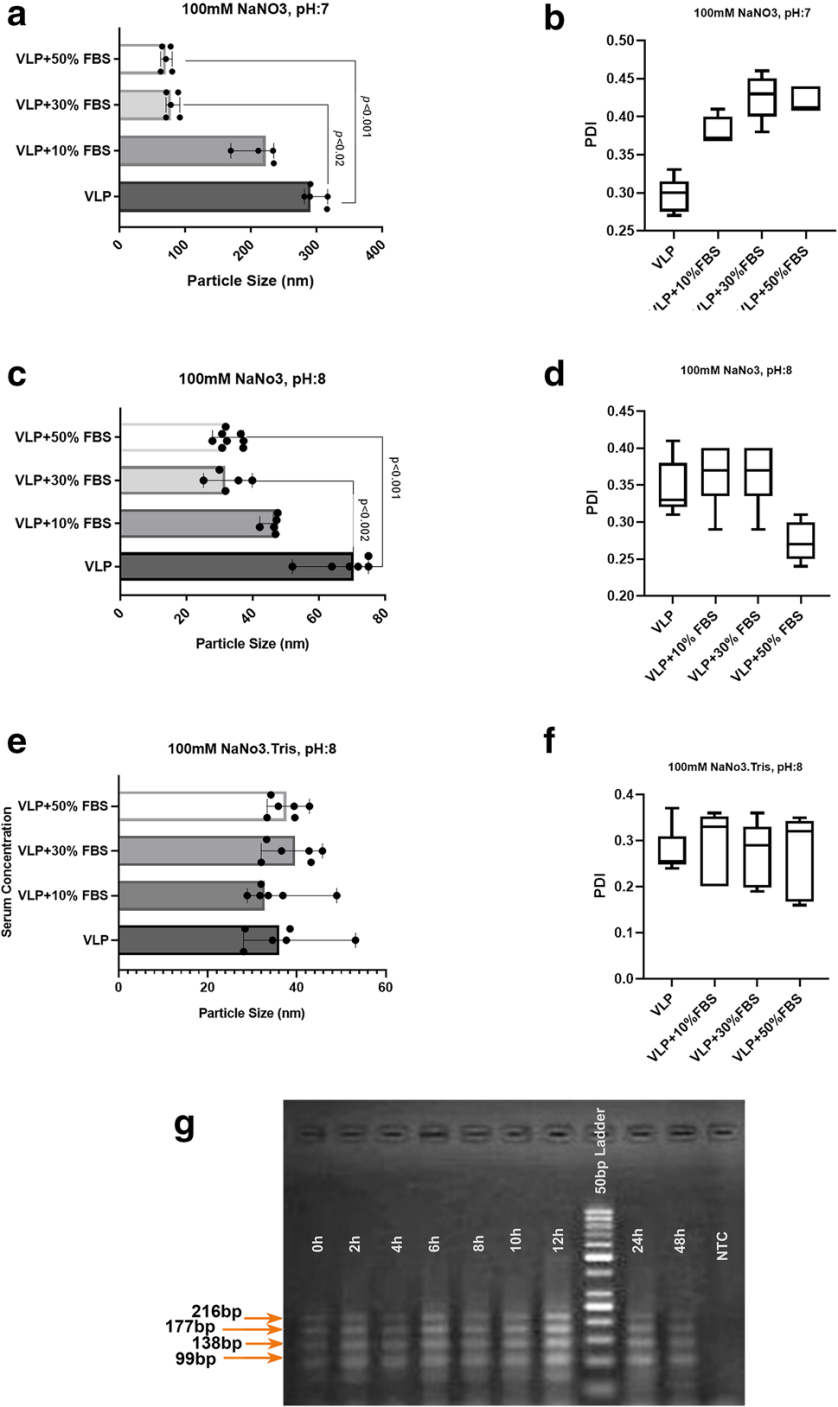
VLPs prepared in NaNO3-Tris buffer were stable in the presence of serum. Size and PDI of particles were evaluated for VLP suspension in different serum concentrations (10%, 30%, and 50%) after 1-h incubation at 4 °C. (**a**,**b**) 100 mM NaNO3 (pH: 7), (**c**,**d**) 100 mM NaNO3 (pH: 8), (**e**,**f**) 100 mM NaNO3-Tris (pH: 8). Results are shown as median with range, p < 0.02, p < 0.001, p < 0.002. (**g**) Gel electrophoresis for RT-PCR fragments of shRNAs extracted from VLP samples that were incubated at 37 °C for 0 h, 2 h, 4 h, 6 h, 8 h, 10 h, 12 h, 24 h, and 48 h time points in NaNO3-Tris (pH: 8) and 50% of Serum.

To make an environment similar to in vivo condition and in favor of further using VLPs in pharmaceutical studies, MS2 VLP suspensions produced in NaNO3 (100 mM, pH: 7 and 8) solutions and NaNO3-Tris (100 mM, pH:8) buffer were incubated with different serum concentrations (10%, 30%, and 50%). While VLPs in NaNO3 (100 mM, pH:7) had a bigger size than VLPs in the same buffer with pH:8, by rising serum concentrations, a decreasing trend of VLP size was observed which was an indicator of serum effects on PEG dispersity. In contrast, the size of VLPs prepared in NaNO3-Tris (100 mM, pH:8) buffer was stable in different serum concentrations (Fig. 5a, c, e, and Supplementary Fig. S4a, b, c). It has been shown that PDI values below 0.7 provide particle size distribution in the midrange^38,39^. Although the PDI values in all three experiments were below 0.45 and, in the midrange, the effect of different concentrations of serum on PDI variation was evident in NaNO3 (100 mM, pH:7, and 8) solutions (Fig. 5b and d). These changes did not occur for the VLPs produced in NaNO3-Tris (100 mM, pH:8) buffer, with values around 0.3 which are acceptable for drug delivery (Fig. 5f).

RNA extraction and RT-PCR experiments for VLP samples generated in NaNO3-Tris buffer (pH:8) in the presence of 50% serum concentration revealed the existence of RNA sequences and their stability up to 48 h at 37 °C (Fig. 5g).

### VLPs prepared in NaNO3-Tris buffer can efficiently enter the cells

**Figure 6:**
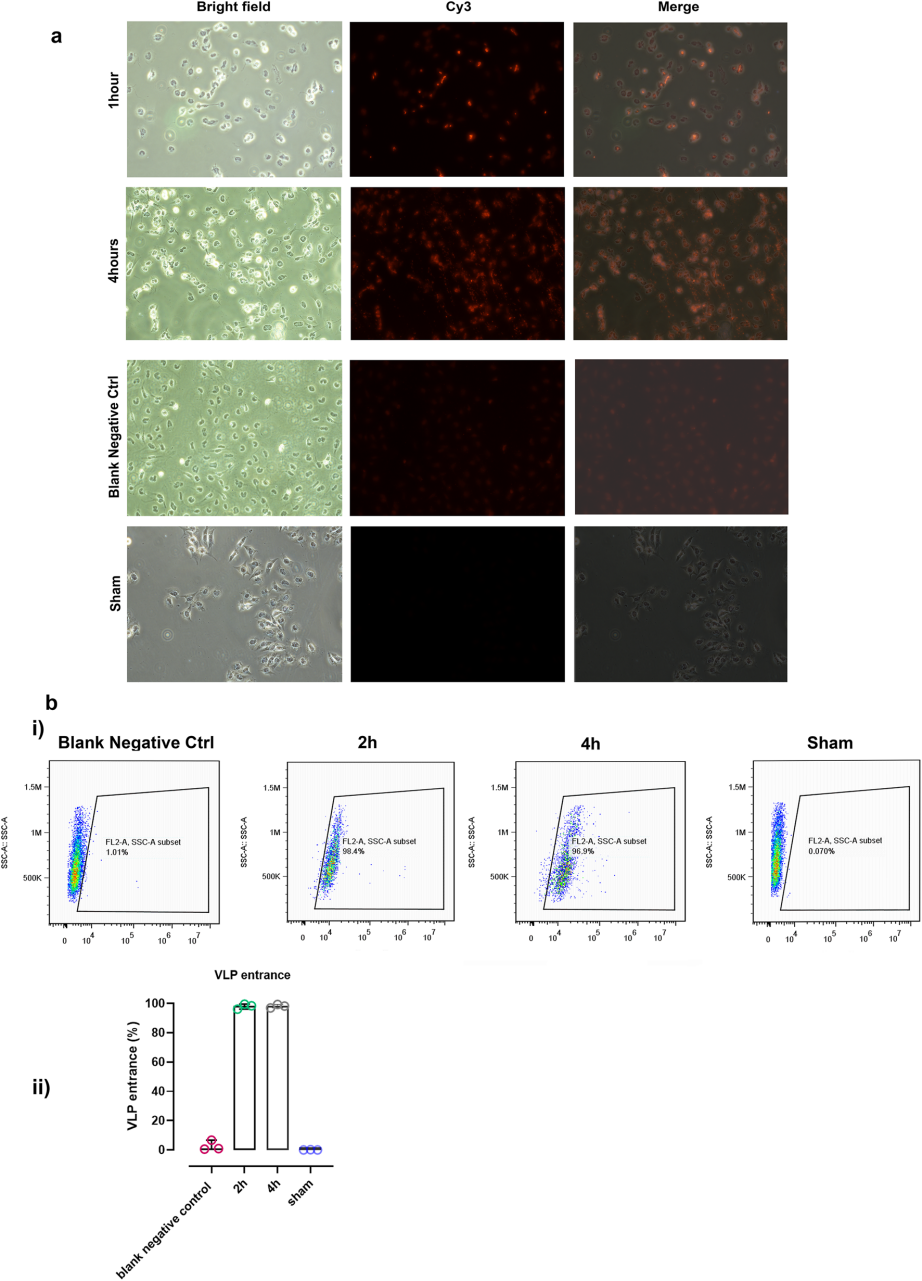
VLPs prepared in NaNO3-Tris buffer can efficiently enter the cells. Neuro2A cells were treated with VLPs prepared in NaNO3-Tris Buffer (0.05 µg/ml). (**a**) Cells were visualized using fluorescent microscopy (Nikon, Japan) in four groups: 1 h and 4 h after VLP treatment, sham (without VLP), and blank negative control (with VLP treatment and without primary antibody) at 100× magnification. Immunocytochemistry images and flow cytometry demonstrated that VLPs (stained with anti MS2 coat protein and secondary-cy3 conjugated antibodies) were internalized by Neuro2A cells effectively. (**b**) i) Intensity plot of cy3 labeled Neuro2A cells in four groups 1 h and 4 h after VLP treatment, sham (without VLP), and blank negative control (with VLP treatment and without primary antibody). ii) diagram representing the average of triplicate results (percentage of the cells with VLP). Data were shown as median with range.

To determine whether the VLPs prepared in NaNo3-Tris buffer could transduce the cells, we used of Neuro2A cell line as immature neuronal cells (neuroblastoma) which could not transfect easily. Immunocytochemistry and flow cytometry was then performed using the MS2 bacteriophage coat protein antibody. Bright intense red spots seen after 1 h, were then dispersed in the cytoplasm after 4 h, indicating the VLP entrance and probable disassembly of the VLPs over the time (Immunocytochemistry, Fig. 6a). The percentage of Cy3 labeled cells was assayed by flow cytometry which revealed the cell-penetrating activity of VLPs after 1 and 4 h (98.4 and 96.9%) effectively (Fig. 6b).

## Discussion

The MS2 VLP is well known amongst different researchers for its small size and great potential of being a promising gene and drug delivery platform^8,14,15,17,23,37^. In this study, we focused on the optimization of the buffers which are used in the precipitation and purification process of VLPs. The ability of different cargos to be encapsulated in VLPs without time-dependent degradation is of great importance^4^. Thus, the most appropriate buffer and its characteristics including pH and ionic strength were identified. Our results led to a new VLP preparation strategy using NaNO3-tris (100 mM, pH:8). This buffer greatly enhanced the consistency (Fig. 4), homogeneity (Fig. 5e and f), and half-life of VLPs (Fig. 4d).

Along with VLP synthesis, various purification strategies such as dialysis^2,9,31^, density gradient by cesium chloride^2,4,8,40,54^, or sucrose^2,41^, and PEG precipitation methods have been examined by different researchers. In this study, we used PEG for VLP purification to avoid probable virus aggregation which has been reported in some examples of the CsCl purification method^42,43^. Indeed, between all the different purification protocols, polyethylene glycol (PEG) precipitation^44^, is the less-toxic and low-cost method, appropriate for purifying proteins from various protein sources^9,45^. In addition, the presence of this uncharged polymeric layer on the outer periphery of the particles leads to an electrostatic and hydrodynamic screening of the negatively-charged inner RNA content of MS2 VLPs, which reduces the effect of internal constituents on the electrophoretic mobility of the particles^2^. Previous studies have shown that various physicochemical factors can affect the capsid stability and aggregation of VLPs^2,9,12,29,30,33^. Among these, pH and ionic strength are the most important factors. Na^+^ containing solutions are used as the most acceptable ionic solutions in the PEG precipitation method^2,9,46^, considering the fact that in monovalent electrolytes such as NaCl, KCl, and LiCl, aggregation of MS2 does not occur within a reasonable kinetic time frame, and MS2 was stable even at high salt concentrations^35^. Thus, we used the NaNO3 solution to prepare an appropriate ionic environment. The shape of VLPs was analyzed by the TEM method. Furthermore, the particle size and PDI were measured as pharmaceutical characteristics of particles that influence endocytosis and cellular uptake.

Irrespective of the purification method, the preparation of MS2 VLPs in two concentrations of NaNO3 solution (1 mM and 100 mM) in different ranges of pH (2 to 7) revealed higher stability of VLPs in 100 mM NaNO3 (pH:7) because of its high electrolyte concentration^2^, pH effectiveness^2^, and minimum mobility^46^. Despite the previous studies showing the stability of virus nanoparticle suspension against aggregation in ≤ 1 M NaCl for more than 10 h^35^, in this study, the same assessment of two concentrations at the stable pH:8 resulted in different sizes of VLPs (Fig. 1). While the VLPs in 100 mM NaNO3 solution (pH:8) were smaller than those VLPs in 1 mM NaNO3 solution (Fig. 1a and c, p < 0.001), PDI of these particles in both concentrations (Fig. 1b) were in the moderate range (0.1–0.3) which could be more acceptable for drug delivery. These results were then in line with the electrostatic properties of the VLPs. Although the surface charge of particles is commonly evaluated via their zeta potential, numerous studies have now demonstrated that definition of this surface charge for complex biosystems such as bacteria^47^ and viruses^48^, as soft bioparticle colloids, has limited application^46,49–51^. The electrophoretic mobility of soft particles such as viruses reaches a non-zero plateau value at large electrolyte concentrations while that of hard colloids tends to zero under similar saline concentrations where particle electrostatic charge is screened by media ions^52^. As extensively discussed by Dika *et al*.^51^, while the electrophoretic mobility of MS2 is systematically negative, due to the negative effective inner charge of the viruses^9,46,50^, the magnitude of electrophoretic mobility (*µ*) decreases with increasing salt concentration as a result of the screening of the virus charge by the ions present in the electrolytic medium, tending to non-zero plateau value for solutions ionic strengths above 100 mM. This latter further emphasizes the better properties of using 100 mM NaNO3 to be used for VLP purification. This negative charge of the particles on the other hand, minimizes the likelihood of aggregation as a result of significant repulsive electrostatic interactions^53^.

The pH of the NaNO3 solution and concentration of ions drastically affects the stability and aggregation of particles. The pattern of these changes for MS2 bacteriophage is completely different from that of MS2 VLP^9^. VLPs produced in 100 mM NaNO3 at pH,8 displayed a smaller size (*p* < 0.0001) compared to VLPs produced at pH,7 (Fig. 1d). However, even in 100 mM NaNO3 solution (pH: 8) with a smaller size, the size of these VLPs (median: 69.28 nm) is larger than MS2 bacteriophage (27–30 nm). Observation of larger VLPs might have resulted from the presence of PEG on the surface of VLPs. 35 days after VLP preparation in 100 mM NaNO3 solution (pH:7), an electron microscopy assay revealed the presence of isolated and uniform VLPs, while immediately after preparation, VLPs were aggregated (Fig. 1f and g) . Since MS2 VLPs are stable within a narrow pH range^2,9,29,31,43^, a feasible explanation for VLP dissociation over time could be the decreasing trend of pH of NaNO3 soluti on from 8 to 5.5 (Fig. 4d and 1f and g).

Tris (pH:8), HEPES (pH:7.2–7.4), and PBS (pH:7.4) buffers, alone or mixed with some electrolytes and chemicals such as MgSO4, NaCl, and EDTA in different molarities have been chosen in various protocols as a protective buffer for VLP preparation and storage^8,24,26,38,42,45^. Applying these buffers for MS2 VLP synthesis determined that the VLPs in Tris Buffer (pH:8) are more uniform and smaller than VLPs in HEPES and PBS buffers (Fig. 2). Despite the uniformity of VLPs in Tris Buffer, these VLPs due to incomplete disassembly or inappropriate assembly are smaller (median: 19.96 nm) than MS2 bacteriophage (27–30 nm). The dimeter of VLPs in the HEPES buffer at pH values of 7.4 and 8 are different. While the particles were larger than VLPs in Tris and PBS buffers at pH:7.4, the particle sizes were undetectable at pH:8 probably due to agglomeration of the VLPs (Fig. 2).

Considering the pivotal role of monovalent cations in VLP formation and stability^35^ and to improve the VLPs’ synthesis process, we employed the new VLP formation strategy by mixing Tris, HEPES, and PBS buffers with NaNO3 (100 mM final concentration). VLPs in Tris buffer (1 M, pH:8) containing NaNO3 (100 mM) displayed a similar size to MS2 bacteriophage which is the convenient size for delivering, whereas VLPs in the same buffer at pH: 7.4 showed the maximum size among all the newly developed buffers (Fig. 2d). Interestingly, adding NaNO3 as an electrolyte to the buffer decreased the size of VLPs, but did not cause a considerable inhomogeneity (Fig. 2e and f). Adding CaCl_2_ to the aqueous suspension of MS2 bacteriophage leads to the aggregation of virus nanoparticles^35^.

In line with the VLPs’ time-lapse stability experiments, previous studies have determined that particles are stable just for 3–4 weeks in 10 mM HEPES and 100 mM NaCl^55^, and remain stable in PBS for > 3 months^8^ at 4 °C. Another study has shown that the stability period of MS2 VLP is at least 4 months at -20 °C in PBS^29^. While VLPs in NaNO3 solution (100 mM) are stable for 3 months at 4 °C, preparation of VLPs in NaNO3-Tris buffer (100 mM, pH: 8) can increase the stability period to more than 12 months (Fig. 4d). Protein concentration is a reasonable marker for evaluating VLP stability. Contrary to VLPs prepared in NaNO3 solution which were degraded over time, protein concentration remained invariable for 12 months in NaNO3-Tris buffer (100 mM, pH: 8) (Fig. 4a). The increased stability of VLPs makes them a suitable candidate to be used in vaccination and drug delivery.

VLP’s behavior and stability in serum are the most critical parameters for delivery. A previous study has shown that MS2 VLPs kept in serum at 4 °C display the best stability for 3 days^29^. On the other hand, the stability of some VLPs in the serum-containing medium at 37 °C for 2 h does not affect VLP’s aggregation or disassembly^56^. Increasing serum concentration in VLP suspension up to 50%, inhibited VLPs from agglomeration. Although the reduction in VLP’s diameter happens probably via serum protein interaction with VLP’s coat protein or PEG ‘s removal, increasing serum concentration of VLPs suspension in NaNO3-Tris (100 mM, pH: 8) did not affect the size. Moreover, in the presence of 50% serum at 37 °C, the shRNA sequence inside the VLPs was protected against degradation for 48 h (Fig. 5g).

These results besides the other characteristics of MS2 VLPs prepared and stored in NaNO3-Tris (100 mM, pH: 8), and also the reliable cell entry properties (Fig. 6), represent MS2 VLP as a good candidate for gene and drug delivery. Therefore, any attempt at optimizing its generation and enhancing the physicochemical properties would be very helpful. Our results demonstrate that using NaNO3-Tris (100 mM, pH: 8) buffer in the process of MS2 VLPs formation, substantially increases the quality and stability of the generated VLPs.

## Material and methods

No animal was used in this study.

### MS2 VLP Plasmid construction and expression system

MS2 VLP expression vector was prepared by amplifying the MS2 Coat and a part of maturase DNA sequences from the pMS27 Plasmid (BCCM/LMBP plasmid Collection, Cabri, Belgium) (Table 1)^18^. The amplified sequence was inserted in-frame into pACYCDuet plasmid (Novagen, Gibbstown, NJ, USA) through BamH1 and HindIII restriction enzyme (Thermo Fisher Scientific Inc., USA) sites. The oligonucleotide sequence encoding the shRNA sequence was designed by siRNA Wizard ™software (Invivogen, USA) (Table 1). The synthesized shRNA (Macrogen Inc., South Korea) fused to four repeats of MS2 bacteriophage *pac* sequence (Macrogen Inc., South Korea) was sub-cloned into the other multiple cloning sites of pACYCDuet plasmid through BglII and Kpn1 restriction enzyme (Thermo Fisher Scientific Inc., USA) sites (Fig. 4e). This sequence (shRNA-4pac, Table 1) was used as a control RNA sequence with the ability to be packed into VLP in each step, plasmid construction was verified by Sanger sequencing (Macrogen Inc., South Korea) and was named the SC Duet.

**Table 1:** Primers and oligonucleotides used in this study.

Name	Oligonucleotide sequence (5′–3′)	Products (bp)	Application
MS27.Fw MS27.Rev	CGGGATCCTGGCTATCGCTGTAGGTAGCC CCCAAGCTTATGGCCGGCGTCTATTAGTAG	1680	amplification of MS2 bacteriophage coat protein sequence
shRNA	GCCTACTCGAACCGTTGATTTCAAGAGAATCAACGGTTCGAGTAGGC	-	Checking the stability of VLPs
shRNA-4pac (sense)	AGATCTGGATCCGGCCTACTCGAACCGTTGATTTCAAGAGAATCAACGGTTCGAGTAGGCCATATGTAA CGATCGTAATTGCCTAGAAAACATGAGGATTACCCATGTCTGCAGGTCGACTCTAGAAAACATGAGGAT TACCCATGTCTGCAGGTCGACTCTAGAAAACATGAGGATTACCCATGTCTGCAGGTCGACTCTAGAAAAC ATGAGGATTACCCATGTCTGCAGTATTCCCGGGTTCATTTACGTACTAGCATAACCCCTTGGGGCCTCTAA ACGGGTCTTGAGGGGTTTTTTGGGTACC	-	shRNA Packaging in the VLPs
shRNA-4pac (anti-sense)	CCAAAAAACCCCTCAAGACCCGTTTAGAGGCCCCAAGGGGTTATGCTAGTACGTAAATGAACCCGGGA ATACTGCAGACATGGGTAATCCTCATGTTTTCTAGAGTCGACCTGCAGACATGGGTAATCCTCATGTTTTC TAGAGTCGACCTGCAGACATGGGTAATCCTCATGTTTTCTAGAGTCGACCTGCAGACATGGGTAATCCTC ATGTTTTCTAGGCAATTACGATCGTTACATATGGCCTACTCGAACCGTTGATTCTCTTGAAATCAACGGTT CGAGTAGGCCGGATCCA	-	shRNA Packaging in the VLPs
VLP.Fw VLP.Rev	CCTACTCGAACCGTTGATTTCAAGAG TGCAGACATGGGTAATCCTCATG	99, 138, 177,216	Checking the stability of VLPs

The SC Duet plasmid was introduced into the BL21(DE3) strain of *E. coli* Bacteria as a prokaryotic expression system. The *Ecoli* BL21(DE3)-SC Duet was cultured in a terrific broth medium at 37 °C, supplemented with 34 µg/ml chloramphenicol (pACYCDuet antibiotic selection). The expression of the sequences was induced by 1 mM IPTG (isopropyl β-d-1-thiogalactopyranoside, Thermo Fisher Scientific Inc., USA) at an OD _600_ = 0.6 for 16 h at 22 °C. Bacteria were precipitated by centrifugation (6000 g, 20 min, 4 °C), and the derived precipitate was resuspended in the appropriate buffer (in 1/5th of the initial bacterial culture volume). Lysozyme treatment (0.05 mg ml^-1^) was performed for 30 min at room temperature followed by sonication (total time: 3 min, 51 W). Cell debris was collected by centrifugation (13500 g, 20 min, 4 °C). The supernatant containing VLPs was filtered through a 0.22 µm membrane (Jet Bio-Filtration Co, China) and stored at 4 °C for further examination^2^. To eliminate cell debris, the suspension was filtered through a 0.1 µm membrane (Sartorius, Germany) and then MS2 VLPs were purified according to the polyethylene glycol (PEG) precipitation method^44^. In brief, the precipitation of VLPs was performed by adding PEG (MW: 6000, Sigma Aldrich, USA) at 10% of the final concentration (w/v) and 500 mM NaCl. The suspension was incubated at 4 °C for 18 h and subsequently was centrifuged at 10000 g for 1 h, 4 °C. The pellet containing MS2 VLPs was resuspended in the buffer used in the first step of purification (in 1/10th of the initial resuspension volume). Eventually, this MS2 VLP suspension was filtered through a 0.22 µm membrane (Jet Bio-Filtration Co, China). All MS2 VLP samples were kept at 4 °C. Solutions of 1 mM, and 100 mM NaNO3 (Merck & Co., Inc. USA), 1 M Tris (Sigma Aldrich, USA) buffer, and HEPES Buffer (Biowest, France) were prepared fresh, one day before experiments.

### MS2 VLPs disassembly and Western blot analysis

To confirm the expression of VLP coat proteins, purified MS2 VLPs were disassembled by diluting 1:2 in 20 mM cold glacial acetic acid for 1 h at 4 °C. To precipitate the free nucleic acids, samples were centrifugated at 4 °C (16,000*g*, 20 min). Then, we assessed the expression of coat protein by SDS-PAGE (12.5% acrylamide gel). Proteins were transferred to a 0.45 µm nitrocellulose membrane (Bio-Rad Laboratories, Inc, USA). The membrane was blocked with 5% non-fat dry milk overnight at 4 °C and then incubated with a polyclonal Anti-Enterobacteria Phage MS2 coat protein antibody (1:7000 dilution, Millipore Sigma Co. USA) for 3 h at room temperature. Incubation with HRP-labeled secondary antibody (1/18,000 dilution, Abcam, USA) for 1 h was performed to confirm the presence of 14 and 28 k Da bands. The membrane was developed with Pierce™ ECL Western Blotting Substrate (Thermo Fisher Scientific Inc., USA). Signals were detected using the Chemiluminescence Detector system (G:BOX Chemi XT Analyser, SYNGENE, Eur.).

### Transmission electron microscopy

The MS2 VLPs were diluted at 1:4 in the buffer. 10 µl of samples were dropped onto carbon-coated copper grids and then were negatively stained by 2% uranyl acetate. After drying, VLPs were observed by transmission electron microscopy (Leo912AB, Germany) at 120 kV at 4000×, 10,000×, 25,000×, 31,500×, 50,000×, 63,000×, and 80,000× magnifications.

### Particle size, polydispersity index evaluation, Zeta potential evaluation, and electrophoretic mobility

MS2 VLPs were diluted 1:4 in selected buffers and solutions. Particle size and polydispersity index (PDI) were measured by particle size analyzer (Vasco3-Cordouan, France) based on Dynamic Light Scattering (DLS), 657 nm at 25 °C. Samples were analyzed by cumulants analysis of 5 consecutive measurements.

Zeta potential for those samples which were not stored in Buffer was estimated by Zeta potential detector (Zeta compact-CAD, France) and reported as zeta potential (mV) line plot at 25 °C. To ensure reliability, it has been performed at least three times.

Electrophoretic mobility (denoted as *µ*) of MS2 VLPs was measured at 23 ± 1 °C using Zeta compact-CAD (France) as a function of buffer containing different electrolytes at pH 7 and 8 for PEG-precipitated VLPs after filtering via a 0.1 µm membrane to remove all the aggregated particles from the suspension. VLPs were diluted at 1:10 in selected buffers and solutions. Experiments were carried out at least three times.

### pH assessment

The pH of all solutions and buffers were measured by a pH meter (Mettler Toledo Co., Switzerland), one day before the experiment. Evaluations were done in triplicates at 0 h, 3 h, 12 h, 24 h, 7 days, and 1-month time points.

### Protein spectrophotometry and MS2 VLPs measurement

Protein concentration was measured by absorbance at 280 nm (A_280_)-1 Abs in 1 cm (1 mg ml^−1^) (Epoch2™ BioTek, Agilent Technologies, USA). Measurements were performed three times at different time points (1, 2, 3, 4, 7, and 12 months). According to the protein concentration and the molecular mass of each VLP particle (14 kDa per coat protein × 180 coat proteins per VLP = 2520 kDa), the number of VLPs in 100 mM NaNO3 solution and NaNO3-Tris buffer (pH:8) was calculated:

Each VLP molecular mass = 2520 kDa = 4.18e−15 mg.

Number of VLPs (in each milliliter) = protein concentration of VLP suspension (mg ml^−1^)/4.18e−15.

### The stability of MS2 VLP containing shRNA-pac in serum

To determine MS2 VLP’s stability in biological conditions, samples were mixed with fetal bovine serum (Biowest, France) (1:1), incubated in 37 °C for 2, 4, 6, 8, 10, 12, 24, and 48 h, and then stored in − 80 °C for further RNA isolation, reverse transcription, and PCR. Furthermore, MS2 VLPs were also incubated in different serum concentrations (10, 30, 50%) for 1 h at 4 °C and particle size and PDI were evaluated diluting 1:4 with additional buffer.

### RNA extraction, reverse transcription, and polymerase chain reaction

300 ul of MS2 VLP suspension in different solutions or buffers (after purification by PEG precipitation method) were subjected to RNA isolation using the Total RNA Isolation Kit (DENAzist Asia Co., Iran) according to the manufacturer’s instructions. The quantity of RNA was determined by a spectrophotometer (spectroEpoch2™ BioTek, Agilent Technologies, USA). A total of 1 µg RNA was used for cDNA synthesis using random hexamer and MMLV reverse transcriptase (Thermo Fisher Scientific Inc., USA). To identify and check the integrity of shRNA-4pac RNA, polymerase chain reactions were carried out by Taq DNA Polymerase Master Mix (Ampliqon, Denmark) and designed primers (Table 1). Polymerase chain reaction steps were 94 °C for 5 min, followed by 35 cycles of 30 s at 94 °C, 15 s at 70 °C, 15 s at 72 °C, and a final extension period of 10 min at 72 °C. PCR products were run on 2% agarose gel.

### Cell culture and Immunocytochemistry

To confirm the VLP entrance, Neuro2A cells (Mouse Neuroblastoma cell line, ATCC) were cultured in Dulbecco’s modified minimal essential medium (DMEM, low glucose) (Gibco: Thermo Fisher Scientific, USA) containing 10% (v/v) of FBS (Gibco: Thermo Fisher Scientific, USA) and 1% penicillin/streptomycin (Biowest, France) at 37 °C, 5% CO2 and 95% humidity. Cells were grown on poly D-lysine (Sigma Aldrich, USA) coated coverslips in four groups (1 h and 4 h after VLP treatment, sham (without VLP), and blank negative control (with VLP treatment and without primary antibody). Briefly, cells were treated with 0.05 µg/ml of VLP suspension 24 h after seeding. The cells were fixed by 2% paraformaldehyde (w/v) for 10 min at room temperature. Subsequently, after washing with cold PBS, the cells were permeabilized with 0.25% Triton X-100 in PBS) for 10 min at room temperature and blocked with PBS containing 1% BSA. Cells were then incubated with primary antibody (anti- Enterobacteria Phage MS2 coat protein, 1:5000, Millipore Sigma Co. USA) for 3 h at room temperature. The coverslips were washed with cold PBS and then incubated for 1 h at room temperature in darkness with a secondary antibody (Monkey anti-rabbit Cy3 conjugated, 1:200, Jackson ImmunoResearch Laboratories Inc., USA). The coverslips were mounted on slides after washing with cold PBS. Stained cells were visualized using fluorescent microscopy (Nikon, Japan).

### Flow cytometry

The quantitative analysis of VLP entrance to Neuro2A cells was performed by flow cytometry. Cells were cultured as previously described in the immunocytochemistry section. Neuro2A cells were treated with 0.05 µg/ml of VLP suspension 24 h after seeding and prepared for flow cytometry in four groups (1 h and 4 h after VLP treatment, sham (without VLP), and blank negative control (with VLP treatment and without primary antibody). Following the cell incubation with VLP (1 and 4 h) and washing with PBS to remove additional VLPs, the cells were trypsinized and subjected to flow cytometry. Followed by Fixing with 4% paraformaldehyde (w/v) for 30 min at room temperature, permeabilizing by 0.2% Triton X-100 (15 min), and blocking with 0.5% BSA and 2% FBS for 30 min on ice. Subsequently, the cells were incubated with primary antibody solution (anti- Enterobacteria Phage MS2 coat protein, 1:500, Millipore Sigma Co. USA) for 3 h at room temperature and then were incubated for 1 h at room temperature in darkness with a secondary antibody (Monkey anti-rabbit Cy3 conjugated, 1:400, Jackson ImmunoResearch Laboratories Inc., USA). Interval of all steps the cells were washed with 0.5% FBS-PBS (cold, 3 times) and were centrifuged at room temperature (1500 rpm, 4 min). the cells were diluted in PBS and analyzed with BD ™Accuri C6 Flow Cytometer (USA). Results were analyzed using FlowJo software (Version 7.6.1, USA) and were shown as an intensity plot (Fig. 3).

### Statistical analysis

Data analysis was performed by GraphPad Prism (version 8.0.2, GraphPad Software Inc., CA). Results were analyzed using the Mann–Whitney and Kruskal Wallis test analysis.

### Equipment and setting

All charts of pictures were set and labeled in Adobe photoshop (2020) and were saved in TIFF format. TEM microscopic and cell images were used without any change. Imaging for ICC experiments was done by Nikon fluorescent microscope in the same condition for all shots at 10× magnification (NIS-Elements F 4.60.00 64-bit software). Agarose gels are full-length versions and just a little brightness and contrast were changed for the entire gels. A full-length original version of the blot was used. All graphs were designed with GraphPad Prism (version 8.0.2, GraphPad Software Inc., CA).

## Supplementary Information

Supplementary Figures.

## Data Availability

The datasets used and/or analyzed during the current study are available from the corresponding author on reasonable request.

## Abbreviations

PEG: Polyethylene glycol
VLP: Virus-like particle
shRNA: Short hairpin RNA
PDI: Poly dispersity index
TEM: Transmission electron microscopy
RT-PCR: Reverse transcription-polymerase chain reaction
