# Author Correction: Optimizing the synthesis and purification of MS2 virus like particles

**DOI:** 10.1038/s41598-022-12923-w

**Published:** 2022-05-23

**Authors:** Khadijeh Hashemi, Mohammad Mahdi Ghahramani Seno, Mohammad Reza Ahmadian, Bizhan Malaekeh-Nikouei, Mohammad Reza Bassami, Hesam Dehghani, Amir Afkhami-Goli

**Affiliations:** 1 Division of Biotechnology, Faculty of Veterinary Medicine, Ferdowsi University of Mashhad, Mashhad, Iran; 2 Department of Basic Sciences, Faculty of Veterinary Medicine, Ferdowsi University of Mashhad, Mashhad, Iran; 3 Institute of Biochemistry and Molecular Biology II, Medical Faculty of the Heinrich-Heine University, Düsseldorf, Germany; 4 Program in Genetics and Genome Biology, Hospital for Sick Children, Toronto, ON Canada; 5 Nanotechnology Research Center, Institute of Pharmaceutical Technology, Mashhad University of Medical Sciences, Mashhad, Iran; 6 Stem Cell Biology and Regenerative Medicine Research Group, Research Institute of Biotechnology, Ferdowsi University of Mashhad, Mashhad, Iran

Correction to: *Scientific Reports* 10.1038/s41598-021-98706-1, published online 06 October 2021

The original version of this Article contained an error in Figure 4E, where thymine was used in place of uracil. The original Figure 4 and its accompanying legend appear below.

**Figure 4:**
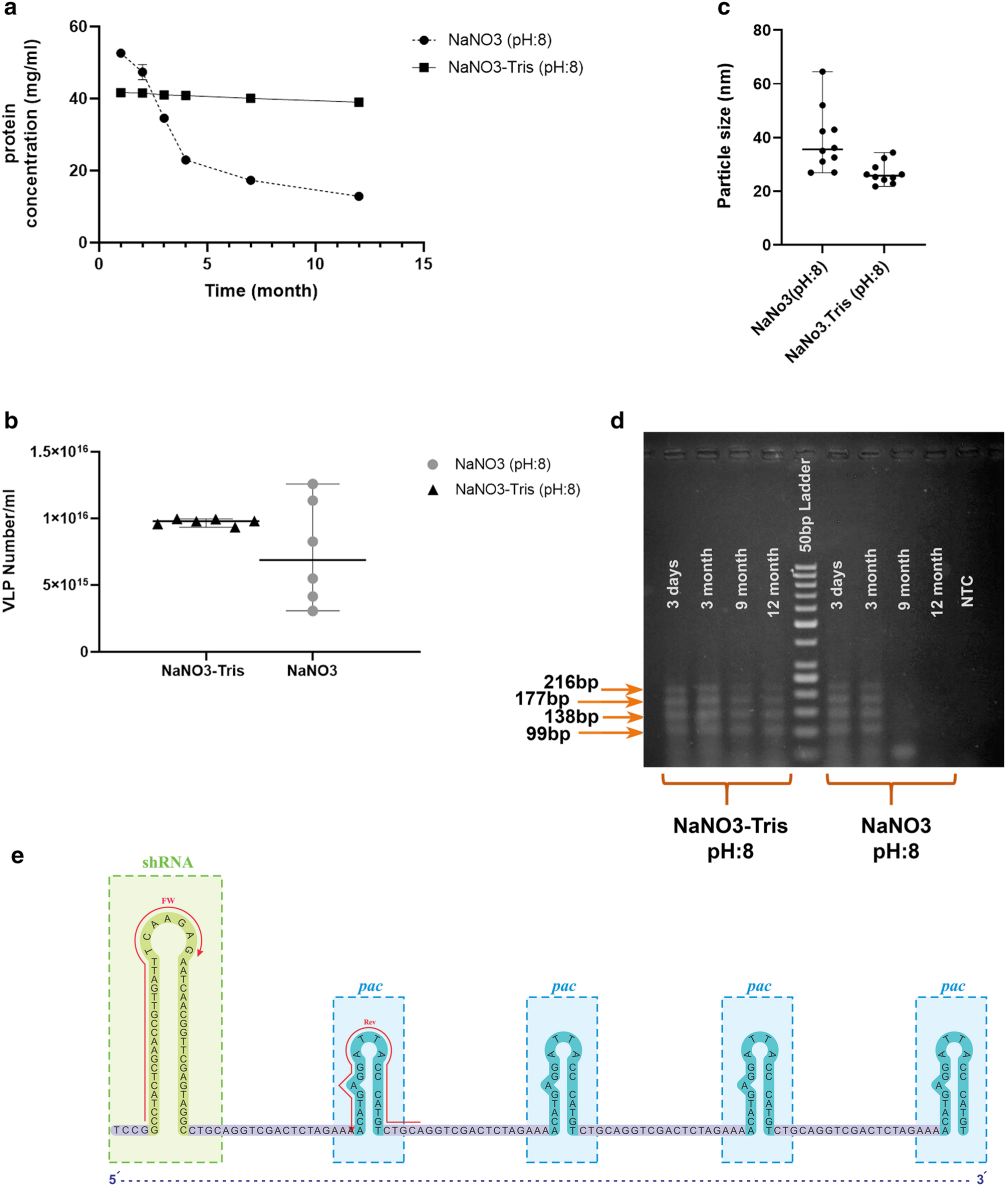
VLPs produced in the NaNO3-Tris were more stable than VLPs produced in the NaNO3 solution. (**a**) Protein concentration evaluation of VLP suspension in two buffers. The amount of protein was reported as mg ml-1. (**b**) Number of VLPs in 1 ml VLP suspension according to the protein concentration. The values are from repeated measurements of samples through 12 months. (**c**) The scattered plot of VLP size in NaNO3 solution and NaNO3-Tris buffer (100 mM NaNO3 and pH: 8, for both of them). The size was analyzed 10 times in each sample and was shown as median with range. (**d**) Gel electrophoresis for RT-PCR fragments (99, 138, 177, and 216 bp); RT-PCR fragments of RNA extracted from VLPs in NaNO3-Tris (pH: 8)- left, and VLPs in NaNO3 (pH: 8)- right, after 3dyas, 3, 9, and 12 months. (**e**) The sequence of shRNA and four hairpins (*pac* sites) was packed in MS2 VLPs. Primer locations were shown as Fw and Rev on the sequence. The stability of VLPs and their ability to protect the inner shRNA was checked by RNA extraction and RT-PCR.

The original Article has been corrected.

