# Supplementary material for: Optimizing the synthesis and purification of MS2 virus like particles
Source: Sci Rep. 2021 Oct 6;11:19851. doi: 10.1038/s41598-021-98706-1 (PMC8494748; doi:10.1038/s41598-021-98706-1)

**Figure S1**

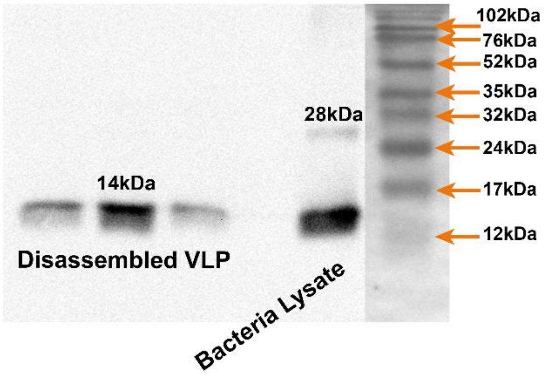


Supplementary Figure 1. Confirmation of coat protein synthesis. For Western blot detection of MS2 coat protein, ~14kDa band was detected after disassembly of MS2 VLPs (three 14kDa bands showed different concentrations of disassembled VLP suspension), while the lysate of the BL21 (DE3) strain of *E. coli* bacteria was confirmed the presence of 14 and 28kDa bands indicating monomers and dimers of the MS2 coat protein.

**Figure S2**

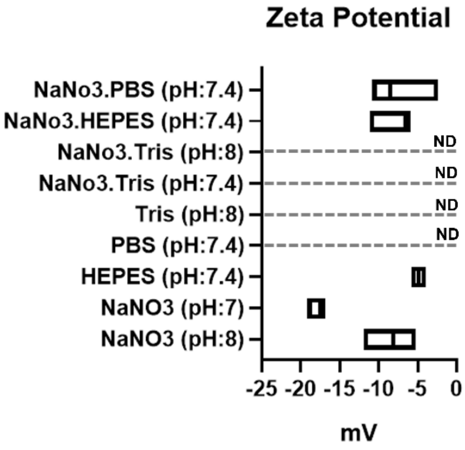


Supplementary Figure 2. Zeta potential of VLPs generated in different solutions. Evaluation of each sample was repeated at least three times and results were shown as zeta potential line plot and median (mV). Because of the nature of buffers, zeta potential could not be estimated for VLP samples in buffers.

**Figure S3**

**VLP size: NaNO3-Tris (pH: 8) vs Tris (pH:8)**

**a**

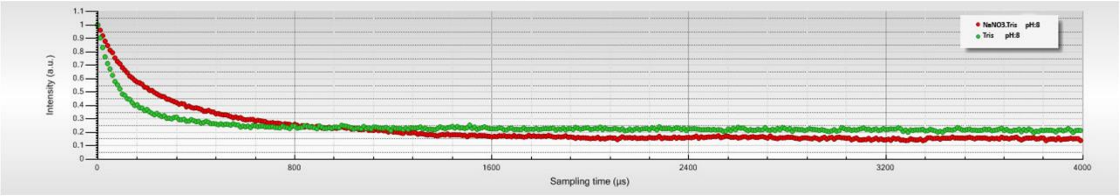


**VLP size: PBS (pH: 7.4), HEPES (pH:8), and HEPES (pH: 7.4)**

**b**

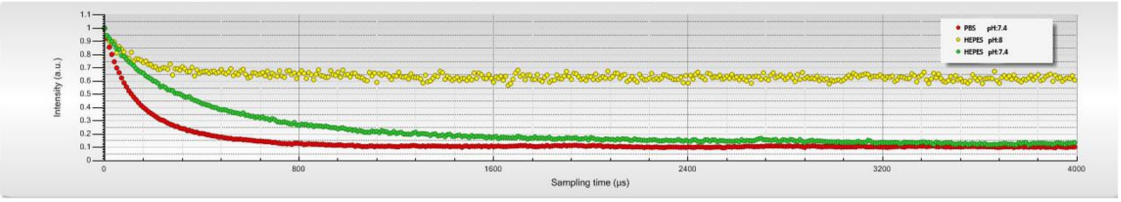


Supplementary Figure 3. Particle size graphs were acquired from VLPs in different buffers. The slope curve bezel of each graph shows the size and homogeneity of particles. The deeper curve protraction and the directed line trail show the small particles and homogenous suspension a) NaNO3-Tris pH:8 vs. Tris pH:8. b) PBS pH:7.4, HEPES pH:8, and HEPES pH:7.4

## Figure S4

**VLP size: NaNO3.-Tris (pH: 8) in serum**

**a**

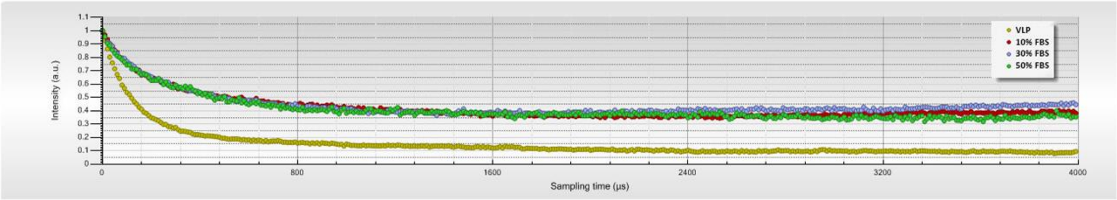


**VLP size: NaNO3 solution (pH: 8) in serum**

**b**

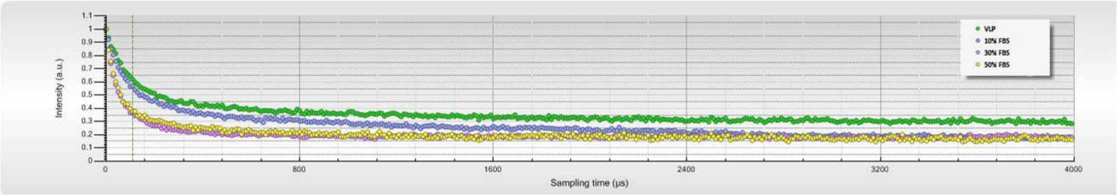


**VLP size: NaNO3 solution (pH: 7) in serum**

**c**

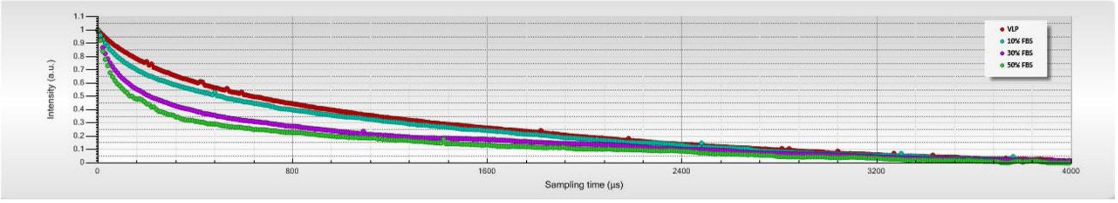


Supplementary Figure 4. The effect of serum concentration on particle size. Serum was added to samples with increasing concentrations (0%, 10%, 30% and 50%) a) VLPs in NaNO3-Tris, pH:8 b) VLPs in NaNO3 solution, pH:8 c) VLPs in NaNO3 solution, pH:7.

## Figure S5

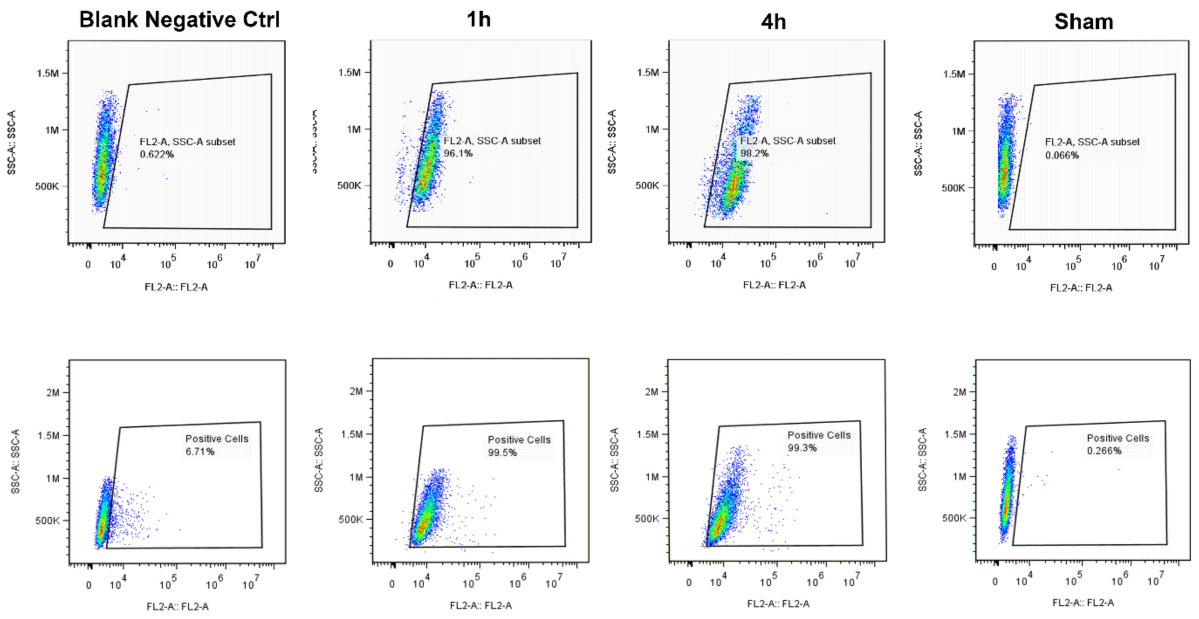


Supplementary Figure 5. VLPs entered Neuro2A cells efficiently (results from the repeated experiment). Intensity plot of cy3 labeled Neuro2A cells in four groups 1hour and 4hours after VLP treatment, sham (without VLP), and blank negative control (with VLP treatment and without primary antibody

## Figure S6: Graphical Abstract

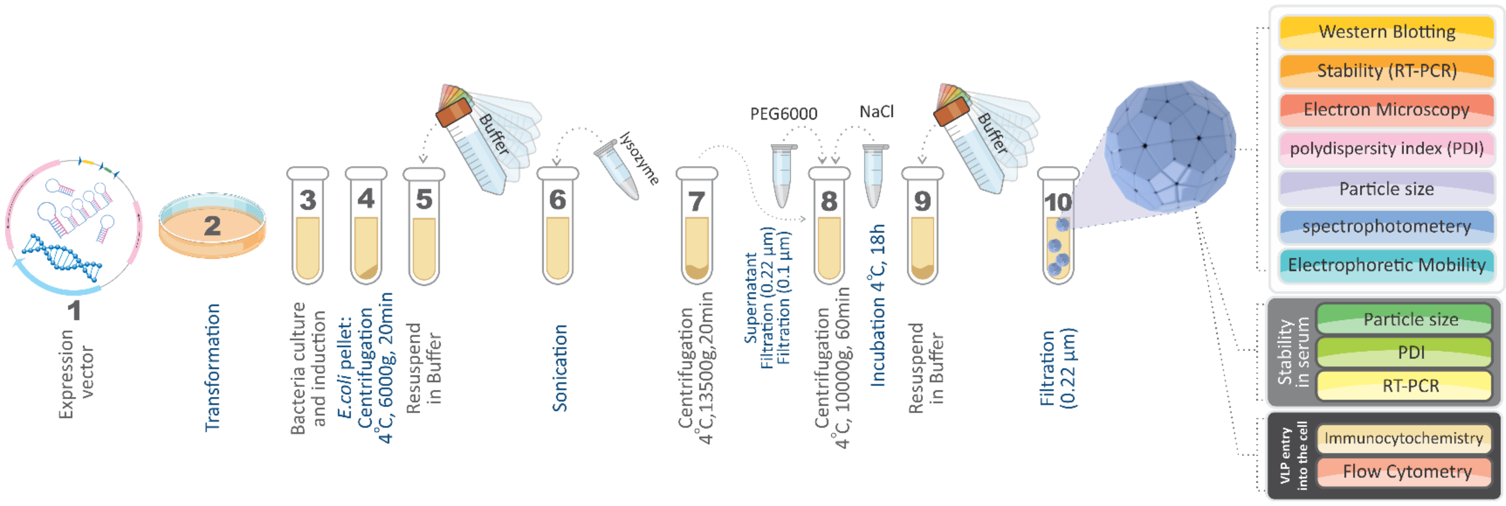

Supplement: Supplementary file 1 — Supplementary Figures. [file 41598_2021_98706_MOESM1_ESM.pdf]
